# Does Forced Plant Maturation by Applying Herbicide with Desiccant Action Influence Seed Longevity in Soybean?

**DOI:** 10.3390/plants12152769

**Published:** 2023-07-26

**Authors:** Larissa Chamma, Gustavo Ferreira da Silva, Samara Moreira Perissato, Cleonei Alievi, Prínscilla Pâmela Nunes Chaves, Valéria Cristina Retameiro Giandoni, Juliano Carlos Calonego, Edvaldo Aparecido Amaral da Silva

**Affiliations:** 1 Department of Crop Science, School of Agriculture, São Paulo State University (UNESP), Botucatu 18610-034, Brazil; gustavo.ferreira@unesp.br (G.F.d.S.); samaraperissato@gmail.com (S.M.P.); prinscilla_@hotmail.com (P.P.N.C.); valeria.giandoni@unesp.br (V.C.R.G.); juliano.calonego@unesp.br (J.C.C.); amaral.silva@unesp.br (E.A.A.d.S.); 2 Department of Crop Science, School of Agriculture, Federal Institute of Goias (IFG), Urutaí 75790-000, Brazil; cleoneialievi@yahoo.com.br

**Keywords:** *Glycine max*, vigor, desiccation, seed quality, germination

## Abstract

Herbicides with desiccant actions may be used to anticipate the harvesting of soybean seeds. This technique aims to minimize the negative influence of biotic and abiotic factors on seed physiological quality at the end of the plant cycle. However, forced seed maturation with the application of herbicides can compromise the acquisition of seed quality components, especially longevity. Thus, the objective of this research was to evaluate the physiological quality of soybean seeds subjected to forced maturation with desiccants. The experiment was performed in a completely randomized design, with a treatment consisting of soybean plants subjected to the application of herbicides with desiccant action at stage R7.3 and another that underwent the natural process of maturation, that is, without herbicide application. The herbicide used was Paraquat. Seed germination, vigor (first germination count, dry mass, seedling length, time to reach 50% germination(t50), emergence index, and emergence speed), and longevity(P50) were evaluated. The herbicides did not affect germination (normal seedlings). However, the acquisition of vigor and longevity, and the preservation of seed vigor during storage were affected. Thus, the results indicate that the application of herbicide with desiccant action interrupts the process of acquisition of seed physiological quality, notably longevity in soybean seeds.

## 1. Introduction

Soybean (*Glycine max* (L.) Merr. (Fabaceae) is a source of protein and oil. The grain contains more protein (40%) than other legume species [1], and can be used as feed by humans and animals [2]. The demand for sources of protein and oil has increased the demand for soybeans in the last decade. Thus, the production of soybean with high-quality seeds is mandatory to expand the cultivation of the species [3,4], and is an important factor leading to crop yield.

For practical reasons, soybean seeds are harvested at the end of the late maturation phase when seed moisture content decreases. Thus, commercial harvesting is delayed until the seeds reach the best moisture content, which reduces the possibility of seed mechanical damage. However, during this period, soybean seeds are highly susceptible to deterioration, since they may experience periods of high humidity and temperatures or prolonged rainfall, which are common in tropical regions [5,6,7,8,9].

Thus, to minimize the possible negative effects of keeping soybean in the field until the seeds reach the moisture content indicated for harvesting, management practices such as the application of herbicide with desiccant action may be used. The herbicides accelerate the drying of the plant, allowing mechanical harvesting of the seeds [10]. Although there are studies on the influence of desiccation products on seed quality, such as germination and vigor, and on chlorophyll retention in soybean seeds [11], no information about the effects on the acquisition of seed longevity is available in the literature.

Seed quality is related to genetic purity, and physical, physiological, and sanitary attributes [12]. However, seed physiological quality is an important factor that possibility to obtain high yields. Seed physiological quality is acquired during seed maturation, which is a process that occurs during seed development when essential components are accumulated.

In soybean seeds, physiological quality is progressively acquired during maturation and late maturation [13]. Germinability is acquired at stage R7.1 (seed coat entirely green and yellow embryo axis). Desiccation tolerance, which guarantees that seed viability will not be affected by the loss of water during maturation, is acquired at stage R7.2 (seed coat mainly yellow with green spots in the middle). Vigor starts being acquired during stage R7.2 and reaches its maximum at stage R7.3 (seed coat completely yellow with a shiny surface and seeds are detached from the fruit). Longevity starts to be acquired at stage R7.2 and progressively increases until the end of late maturation at stage R9 (brown seed coat and dry seeds). Seed longevity is the total time span during which seeds remain viable [13,14,15].

Seed longevity is influenced by seed structure, germination, phenology, and environmental factors, including climatic conditions experienced by the seeds during the post-zygotic phase [16] and other aspects such as temperature and relative humidity during storage [17].

This is a plant survival mechanism, which enables it to survive unfavorable conditions and preserve plant genetic resources [18,19]. Therefore, these facts suggest that longevity is a dominant aspect of the seed, being highly influenced by biotic and abiotic environmental factors, which can affect the seedling establishment and crop yield [20,21]. Seed longevity also affects germination and establishment, and therefore crop yield and quality in agroecosystems through its effect on field emergence rates, and photosynthetic capacity [22].

Thus, we hypothesize that forced maturation on mother plants during seed maturation by using herbicides with desiccant action can impact seed physiological quality, mainly longevity. Therefore, the aim of this research was to study the impact of forced maturation using desiccants on seed physiological quality.

## 2. Results

**Figure 1:**
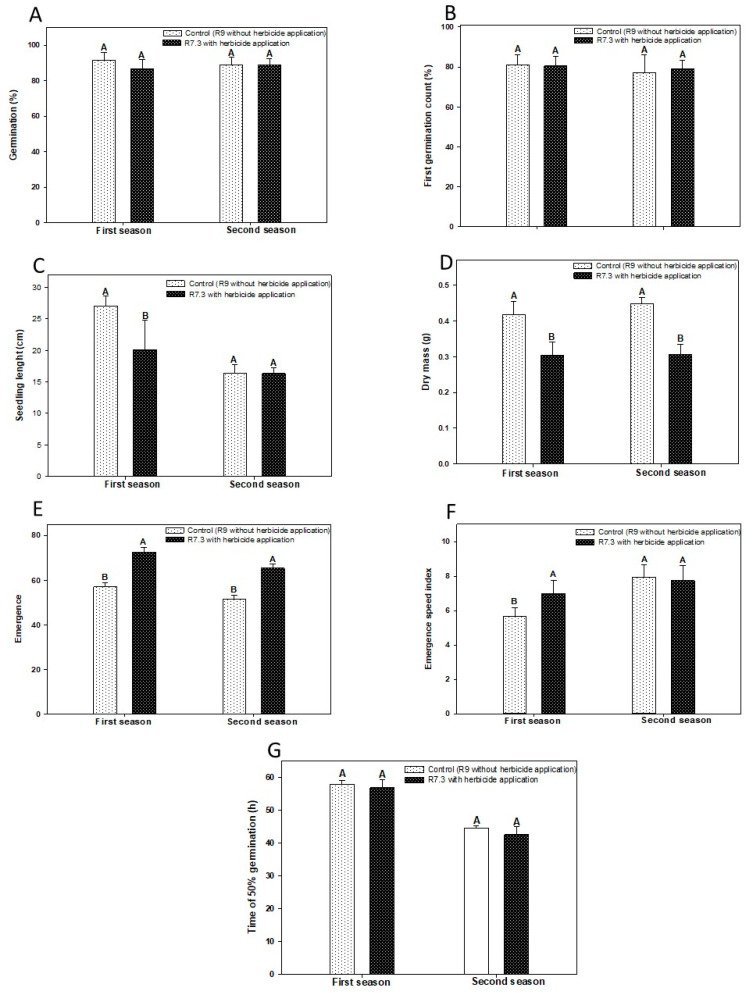
Percentage Mean values of germination (%-(**A**)), first germination count (FCG-%-(**B**)), seedling length (cm-(**C**)), dry seedling mass (g-(**D**)), emergence speed index (ESI-(**E**)), emergence (%-(**F**)) and time in hours for 50% of the seeds to germinate (T50-(**G**)) for desiccated soybeans in 7.3 and control as a function of the desiccant application. This means that the same uppercase letters do not differ in the comparison between the desiccation treatments within the same year of evaluation by the LSD test at 5% of probability.

The seed water content ranged from 9.0 to 9.8%, with no significant differences. In both crop seasons, there were no significant differences in the germination potential of the seeds, such as first germination count (FCG) and time for 50% of seeds to germinate (t50) between treatments with or without the use of desiccant (Figure 1A,B,G). However, the use of desiccant negatively affected seed vigor by reducing seedling length by 26% (only in the first season), and seedling dry mass by 27 and 32% in the first and second seasons, respectively, when compared to treatment R9 without herbicide application. (Figure 1C,D).

The absence of the desiccant also negatively affected some vigor traits, reducing the emergence speed index (ESI) to 19% (Figure 1E), in the first season; and emergence by approximately 21% (first and second seasons) (Figure 1E,F).

**Figure 2:**
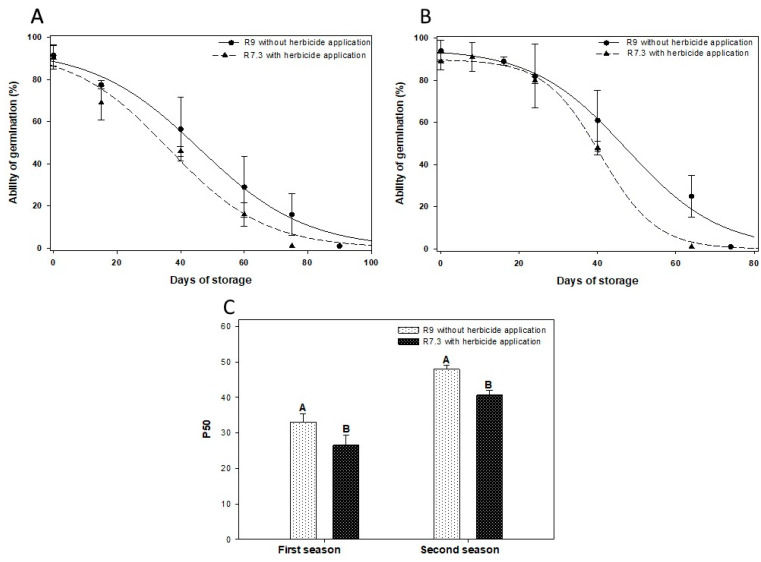
Germination ability over storage time in the first (**A**) and second season (**B**). Time in days to loss of 50% of viability (P50) in the first and second season (**C**) for desiccated soybeans in 7.3 and control. The seeds were stored at a temperature of 35 °C and relative humidity of 75%. The uppercase letters indicated mean differences analyzed by least significant difference (LSD) at 5% of probability.

The study of longevity through the evaluation of P50 shows that both seasons presented reduced longevity for the seeds harvested from plants that went through the forced maturation process (Figure 2). The sigmoid longevity curves are shown in Figure 2A,C. In both crop seasons, the application of herbicide at stage R7.3 reduced the p50 compared to the R9 stage. In the first season, seed longevity decreased. The seed longevity without desiccant application was 20% higher in the first season, and 15% higher in the second season, compared to the treatment with the application.

**Figure 3:**
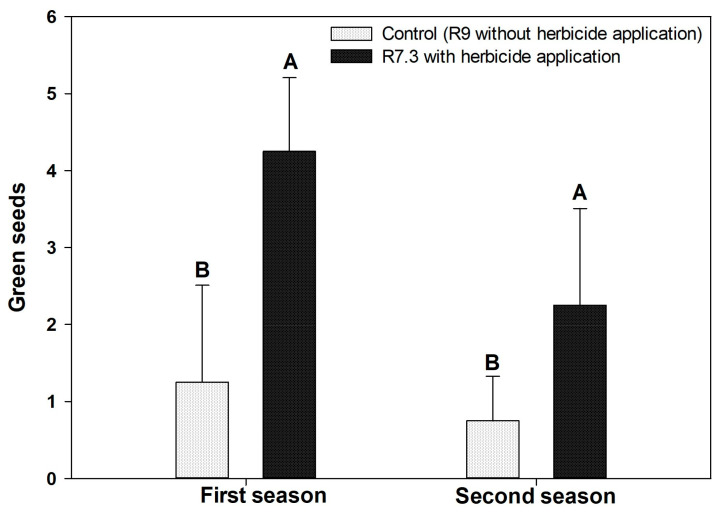
Green seeds (%) in treatments with and without desiccant in the first and second season para for desiccated soybeans in 7.3 and control. This means that the same uppercase letters do not differ in the comparison between the desiccation treatments within the same year of evaluation by the LSD test at 5% of probability.

The incidence of green seeds was superior in the desiccated seed lots in both seasons (Figure 3).

To verify the storage effect on seed physiological quality after a period of approximately 10 months of storage in a cold chamber (10 °C and 40% RH), vigor and germination tests were performed again.

**Figure 4:**
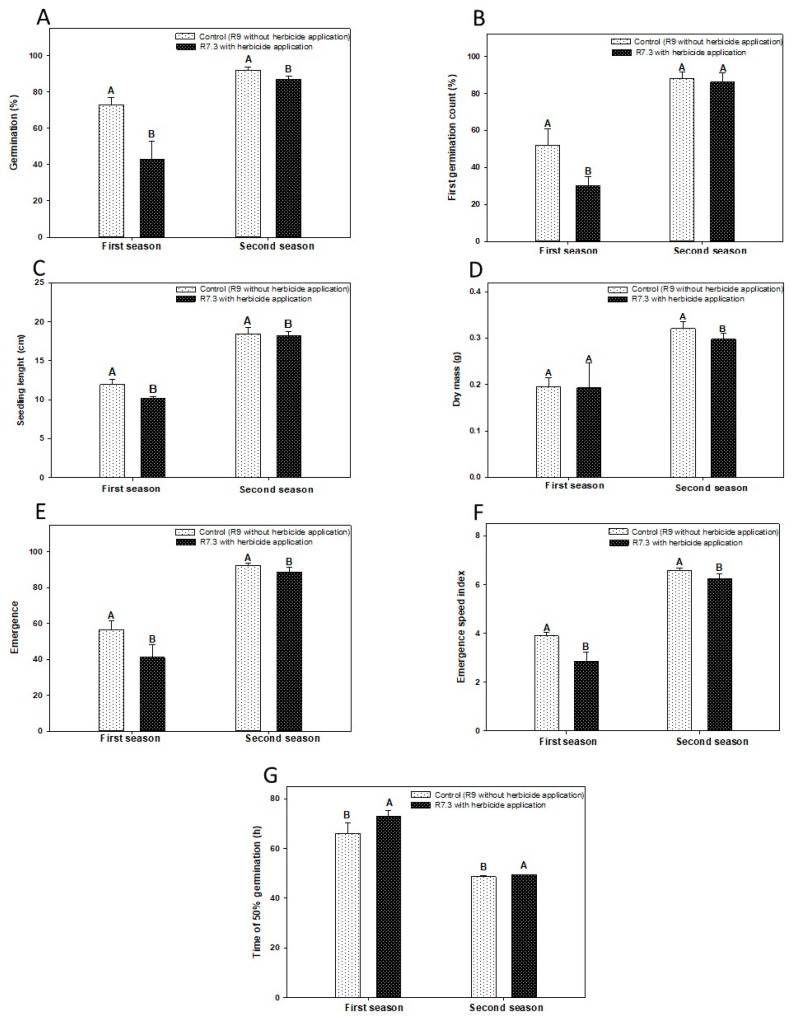
Mean germination values (%-(**A**)), first germination count (FCG-%-(**B**)), seedling length (cm-(**C**)), dry seedling mass (g-(**D**)), emergence (%-(**E**)), emergence speed index (ESI-(**F**)), time for 50% of the seeds to germinate (T50–hours-(**G**)), of soybean seeds as a function of desiccant application after ten months of cold storage for desiccated soybeans in 7.3 and control. Means followed by the same uppercase letters on the line do not differ from each other by the LSD test at 5% of probability.

The desiccant treatment after storage took longer to reach the T50 in both seasons (Figure 4G). This trait was not significantly affected in the seeds evaluated soon after harvest. However, after 10 months of storage, R9 (without herbicide application) reached T50 in seven hours in the first harvest, and an hour in the second harvest (Figure 4G).

The FCG after storage only showed a difference in the first season, with 73% more seeds germinating in the R9 treatment (without herbicide application) (Figure 4B). The dry mass variable differed only in the second season, with seedlings with 8% more mass in the R9 (treatment without herbicide) (Figure 4D).

The germination initially (soon after the harvest) did not present significant differences between the control and treatments, being 5 and 0% for the first and second harvests, respectively (Figure 1A). After the storage period, there was a significant difference of 30 and 5% in the first and second seasons, respectively (Figure 4A).

## 3. Discussion

There is a lack of research on desiccant applications considering the impacts on the acquisition of seed quality (germination, vigor, and longevity). Thus, in this research, we observed that the use of desiccant on soybean affects some parameters during the acquisition of seed physiological quality. Our study demonstrated that germination is not a parameter affected by the application of the desiccant at the R7.3 stage for both seasons studied. However, in relation to vigor, some parameters such as seedling length and emergence speed index, showed a different behavior from one season to another. In the first season, a difference was found between the control and treatments, but in the second season, there were no differences. Possibly, the inheritance of plants in the first harvest could be more uneven due to abiotic or biotic factors; thus, revealing the need for future studies that address the effects of disease and disease management, and soil and environmental effects in relation to the use of desiccants. The acquisition of longevity was also affected by the use of the desiccant tested.

Paraquat, a non-selective contact herbicide, causes desiccation and defoliation in the plant [23]. It acts by inhibiting photosynthesis, accepting electrons from photosystem I, and transferring them to molecular oxygen. In addition, they immediately reduce oxygen to superoxide radicals (O^2−^), which may directly or indirectly cause cell death and increase the drying of grain and foliage of crops [24,25,26]. In this way, desiccation acts to ensure greater uniformity in the harvest, considering that it is impossible for all seeds and plants to be in the same stage at the same time [27,28,29].

The soybean plant presents an uneven maturation process of the pods and even of the seeds inside the pods [30]. Thus, at the time of desiccation, not all the plant pods were at the R7.3 stage, and when going through the desiccation process, there was a stoppage in the transport of photoassimilates necessary to guarantee the physiological quality of the seeds. Furthermore, by accelerating the natural drying process, there is an interruption of the acquisition of components that normally occurs during late maturation, negatively affecting longevity and vigor maintenance throughout storage.

Another process that demonstrated how the desiccant interferes in the acquisition of physiological quality of seeds was the greater retention of chlorophyll in seeds that underwent the desiccation process (Figure 3). As already evidenced by other studies, the chlorophyll retention process is mainly associated with low rainfall and high temperatures during the maturation phase [31]. In addition to climate, pre- and post-harvest practices, such as desiccant applications and premature harvesting followed by drying at high temperatures, also affect the natural process of chlorophyll degradation [11]. Although it is not yet known whether chlorophyll retention is the cause or a marker, what research has reported is that they are associated with reduced germination potential, vigor, and especially seed longevity [31,32].

It is not yet known for sure whether chlorophyll has a direct impact on seed quality, or whether it is a marker of maturation disorders. However, during the seed maturation process, it is expected for chlorophyll degradation to occur naturally [13]. Thus, the increase in the percentage of chlorophyll in dried seeds is an indication of damage to the natural process of seed degradation.

Seed longevity is a feature that has been little studied in soybean seeds, as long periods of storage do not occur in commercial materials. However, seed longevity is a characteristic responsible for maintaining vigor under stress and storage conditions [19]. In this way, studies have demonstrated the importance of the complete acquisition of longevity in soybean seeds in order to preserve their vigor. Ref. [19] observed that the germination speed and loss of elongation capacity of roots and hypocotyls occur simultaneously with the loss of germination ability and are, therefore, good markers of vigor in seedlings.

Research has demonstrated the effect of longevity by assessing the ability to germinate after storage [17,31,33]. Seed vigor is an extremely important factor for crop development and establishment. In this research, we tried to confirm the effect of the loss of vigor during storage using the FCG, T50, seedling length, dry mass, ESI, and emergence tests. The results of this research confirmed that in addition to the loss of germination ability, there is also a decrease in several other parameters of vigor (Figure 1, Figure 2 and Figure 4).

The emergence tests and the ESI were the ones that most evidenced the importance of evaluating vigor after storage. The tests showed there was an inversion of the results. If previously, we observed a behavior in which the treatment with desiccant was superior in relation to the control, after storage, we observed the opposite behavior.

In this research, we verified that the use of desiccant for the forced maturation of the seeds influenced the vigor acquisition, mainly of stored seeds, and also the acquisition of longevity of soybean seeds. Such alterations are due to the fact that after physiological maturity, known as the moment when the seed disconnects from the mother plant, reactions still occur in the seeds resulting in the accumulation of non-reducing sugars, abundant proteins in late embryogenesis and heat shock proteins, which are associated with longevity [33,34,35]. In addition, there is also the accumulation of antioxidants such as glutathione [36], tocopherols [37], and flavonoids present in the forehead [38]. It is clear, then, that longevity is an important characteristic mainly for breeding programs that store genetic materials in germplasm banks for the development of new cultivars. Thus, it is important to guarantee the correct management of the soybean crop, aiming for the maximum acquisition of seed longevity through the careful use of desiccants.

## 4. Materials and Methods

### 4.1. Seed Production

The experiment was performed during the crop season of 2018/2019 (first season) and 2019/2020 (second season), at the experimental area of the Lageado farm belonging to the College of Agricultural Sciences from the University of the State of Sao Paulo, located at the city of Botucatu, State of São Paulo-Brazil, with latitude 22°49′ S, longitude 48°25′ WGrw and altitude of 786 m. According to the Köppen classification, the climate is classified as CWa, a mesothermal climate with dry winter.

The soil is a Rhodic Hapludox type [39] safe, with a clayey texture, and the experiment was performed under a consolidated no-till system (since the year 1986). The chemical [40], physical (Blake and Hartge, 1986; [41,42]) and granulometric (EMBRAPA, 2017) properties of the soil, in the 0.00–0.20 m layer were as follows: pH (CaCl_2_): 4.9; P (resin): 50.9 mg dm^−3^; organic matter: 25.6 mg dm^−3^; H + Al: 44.3 mmol_c_ dm^−3^; Ca: 1.9 mmol_c_ dm^−3^; Mg: 1.4 mmol_c_ dm^−3^; K: 4.5 mmol_c_ dm^−3^; Al: 3.4 mmol_c_ dm^−3^; macroporosity: 0.12 m^3^ m^−3^; microporosity: 0.39 m^3^ m^−3^; total porosity: 0.51 m^3^ m^−3^; bulk density: 1.17 g cm^−3^; penetration resistance: 3.63 MPa; sand: 235 g kg^−1^; silt: 241 g kg^−1^; clay: 524 g kg^−1^.

**Figure 5:**
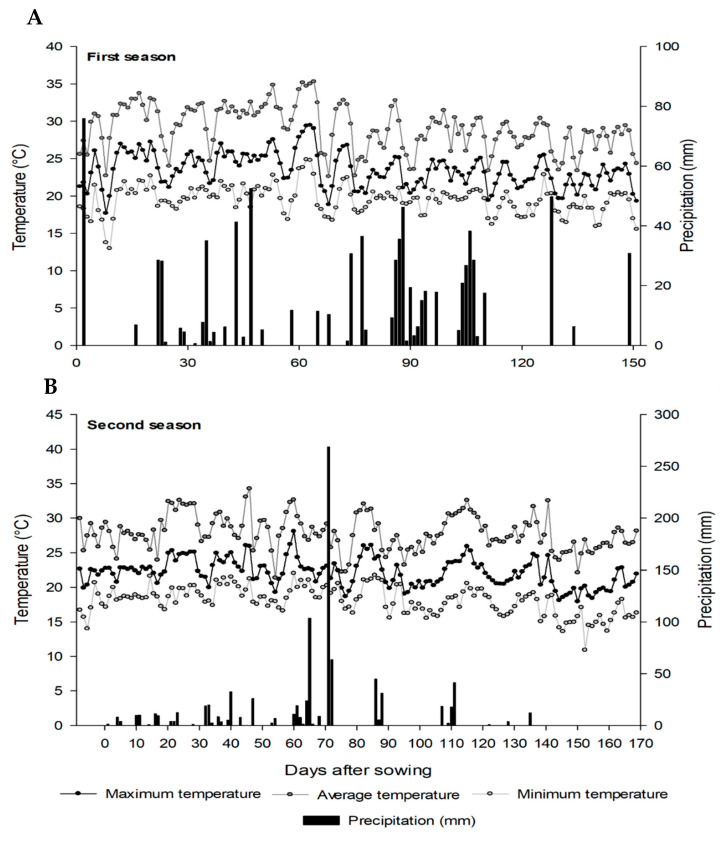
Maximum, average, and minimum temperatures and rainfall during the first and second seasons of soybean. (**A**) First season (2018/2019) (**B**) Second season (2019/2020).

The data of maximum, average, and minimum temperatures and rainfall during the crop seasons of 2018/2019 (A) and 2019/2020 (B) are shown in Figure 5.

### 4.2. Treatments and Experimental Design

The cultivar used was a transgenic commercial cultivar TMG 7062 IPRO. The sowing of the first season was performed on 7 December 2018, and the second season on 8 December 2019. Sowing was carried out at a spacing of 0.45 m between rows, aiming at a density of 300 thousand plants ha^−1^. The seeds used in the treatments and control were treated with Carboxin + Thiran fungicide, Thiamethoxam insecticide, *Bradyrhizobium* sp. inoculant, and cobalt and molybdenum micronutrients, and sowing fertilization was performed with 60 kg ha^−1^ of potassium oxide (K_2_O) and 60 kg ha^−1^ of phosphorus pentoxide (P_2_O_5_), using potassium chloride (KCl) and simple superphosphate.

Soybean phytosanitary management involves weed control with the application of the herbicide Glyphosate (1.8 kg a.i. ha^−1^) associated with the herbicide Sethoxidim (1.25 kg a.i. ha^−1^). The fungicides Pyraclostrobin + Epoxiconazole (0.08 + 0.03 kg a.i. ha^−1^, respectively) and Azoxystrobin + Cyproconazole (0.06 + 0.024 kg a.i. ha^−1^, respectively) and the insecticides Thiamethoxam + Lambda-Cialotrin (0.028 + 0.21 kg a.i. ha^−1^) were applied preventively. Pre-harvest desiccation was carried out using herbicide paraquat (0.4 kg a.i. ha^−1^; 200 L ha^−1^ of spray volume). The desiccant application was carried out with a Jacto Falcon AM14/Vortex sprayer, with flat jet tips (fan) model ADI 11002, without wind and with an air temperature of 20 °C.

The pre-harvest desiccation treatment was performed when more than 76% of the leaves and pods of the plants in the field were yellow [13,19,43]. The seeds were at the R7.3 stage, in which most of the seeds had a uniformly yellowish seed coat, with no sign of chlorophyll, with a shiny surface, and the seeds were already detached from the pod. The seed water content was around 55 ± 1%. In parallel, the control, that is, non-desiccant treatment, underwent natural maturation until reaching R9, known as the harvest point, in which the seeds have a dry appearance and water content below 15% [13,19,43]. Thus, the treatments consisted of seeds from plants at R7.3 (desiccated) and from plants at R 9 stage (control). For desiccation, the herbicide used was Paraquat (0.4 kg a.i. ha^−1^). The characterization of the phenological stages at the time of harvest considered the visual characteristics of the plants and seeds (more than 50% of the crop). The seeds were harvested four days after herbicide application. The pods were harvested and threshed manually, and later the seeds were placed in paper bags and stored in a cold chamber at 10 °C and 40% of relative humidity (RH).

### 4.3. Seed Quality Assessment

The seed water content was evaluated by the oven method at 105 ± 3 °C for a period of 24 h [44], with three replications of 15 seeds. The results were expressed as a percentage of water on a wet basis.

Seed germination was performed with four replications of 25 seeds, using a roll of paper moistened with distilled water equivalent to 2.5 times the paper dry mass, and then the rolls were placed in a biological oxygen demand (BOD) chamber at a constant temperature of 25 °C under the absence of light. The germination percentage was calculated by counting normal seedlings at five (first germination count used as vigor index) and eight days after sowing on paper (total germination). Normal seedlings are those that show the potential to continue their development and form plants that are capable of establishing themselves in the field, showing no absence of essential structure for development (the aerial part with plumule and main root). Abnormal seedlings are those that do not show the potential to continue their development and give rise to normal plants; even growing under favorable conditions, they may show impairment in shoot and root structures [44].

Radicle protrusion verification was performed by placing four replicates of 25 seeds in Petri dishes using three sheets of filter paper as substrate and distilled water equivalent to 2.5 times the dry mass of the filter paper. The Petri dishes were placed in a BOD at a constant temperature of 25 °C in the absence of light. The radicle protrusion was verified every six hours, counting the number of seeds that presented a radicle with two millimeters of length. The time required to reach 50% germination of viable seeds (T50) was calculated by analyzing the cumulative germination data using the curve-fitting model of the Germinator software [45], and the results were expressed in hours.

For the length and dry mass of the seedlings, four replications of 10 seeds from each treatment were used, arranged in a roll of paper moistened with distilled water equivalent to 2.5 times the dry mass of the paper. The seeds were arranged on a line drawn longitudinally in the upper third of the paper, with the seed hilum facing the lower portion of the paper, in order to guide the seedling growth in a straighter line [46]. Paper rolls were conditioned in a BOD chamber at 25 °C in the dark. The average length of seedlings was measured on the seventh day after the installation of the test, with the aid of a ruler graduated in cm. After analyzing the length, the seedlings were kept in an air circulation oven at a temperature of 60 °C to obtain the dry matter mass, and the results were expressed in grams.

Seedling emergence was evaluated by daily counting of seedlings whose cotyledon was open above the soil in the sand at field conditions (exposed to environmental conditions). This evaluation was done until stabilization of the number of seedlings emerged. The emergence speed index (ESI) was obtained using the equation proposed by [47], in which: ESI = E1/N1 + E2/N2 + … En/Nn. Where: E1, E2, … En, refers to the number of emerged seedlings computed in the first, second, and last counts; N1, N2, … Nn refers to the number of days from sowing to the first, second, and last count. At the end of the test, the total number of emerged seedlings was determined.

To evaluate longevity, the seeds were placed in airtight boxes with saturated sodium chloride (NaCl) solution and stored at 35 °C and 75% RH, according to the methodology described by [48]. The tests were conducted until the seeds died. Normal seedlings were evaluated after eitght days of the experiments beginning. The germination conditions used were the ones described previously for the germination experiments. From the results, the sigmoidal curve for each treatment was obtained, and seed longevity was expressed in P50 (time in days to loss of 50% of viability) [19].

Green seeds were visually determined using four replications of 100 seeds by considering any green pigmentation observed, and the results were expressed in percentage [31].

After a period of approximately 10 months of seed storage in a cold chamber, the treatments were evaluated again for germination and vigor (first count, T50, emergence, ESI, dry mass, length).

### 4.4. Data Analysis

The data were subjected to the Kolmogorov–Smirnov test to verify the normality of the data and equality of variance. When the differences among the data can be significant, the ANOVA was performed. Mean differences were discriminated by least significant difference (LSD) at 5% of probability.

## 5. Conclusions

The process of forced maturation using desiccants on mother plants does not affect germination. However, seed vigor and vigor during storage are affected, showing that the application of desiccants compromises the acquisition of seed physiological quality, notably longevity.

## Data Availability

Not applicable.
